# Lithotomy

**Published:** 1831

**Authors:** 


13. *Lithotomy.*-Mr. Rigal, of the Royal Academy of Medicine, (Paris,) recently extracted a stone from the bladder of a girl, aged 20 years, which weighed ten ounces and five drachms; it filled the bladder, and was removed by the vagino-vesicale operation. In another case he performed the bilateral operation, and met with two adherent sacs, the extraction of which caused a rent in the bladder; the hemorrhage which ensued was arrested by injections of very cold vinegar and water. The patient recovered. The same operation was performed on a patient aged 55, who died on the fifth day after the operation. The bladder was completely filled with three very large stones, which could not be seized for extraction. M. Amussat suggested, notwithstanding the hypertrophy of the bladder, that it would have been better to have performed the hypogastric operation.

